# How Do Transformers Model Physics? Investigating the Simple Harmonic Oscillator

**DOI:** 10.3390/e26110997

**Published:** 2024-11-19

**Authors:** Subhash Kantamneni, Ziming Liu, Max Tegmark

**Affiliations:** Institute for Artificial Intelligence and Fundamental Interactions, Massachusetts Institute of Technology, Cambridge, MA 02139, USA; zmliu@mit.edu (Z.L.); tegmark@mit.edu (M.T.)

**Keywords:** mechanistic intepretability, AI for science, physics

## Abstract

How do transformers model physics? Do transformers model systems with interpretable analytical solutions or do they create an “alien physics” that is difficult for humans to decipher? We have taken a step towards demystifying this larger puzzle by investigating the simple harmonic oscillator (SHO), $\ddot{x}+2\gamma \dot{x}+\omega_{0}^{2}x=0$, one of the most fundamental systems in physics. Our goal was to identify the methods transformers use to model the SHO, and to do so we hypothesized and evaluated possible methods by analyzing the encoding of these methods’ intermediates. We developed four criteria for the use of a method within the simple test bed of linear regression, where our method was y=wx and our intermediate was *w*: (1) Can the intermediate be predicted from hidden states? (2) Is the intermediate’s encoding quality correlated with the model performance? (3) Can the majority of variance in hidden states be explained by the intermediate? (4) Can we intervene on hidden states to produce predictable outcomes? Armed with these two correlational (1,2), weak causal (3), and strong causal (4) criteria, we determined that transformers use known numerical methods to model the trajectories of the simple harmonic oscillator, specifically, the matrix exponential method. Our analysis framework can conveniently extend to high-dimensional linear systems and nonlinear systems, which we hope will help reveal the “world model” hidden in transformers.

## 1. Introduction

Transformers are state-of-the-art models for a range of tasks [1,2,3,4], but our understanding of how these models represent the world is limited. Recent work in mechanistic interpretability [5,6,7,8,9,10,11,12] has shed light on how transformers represent mathematical tasks like modular addition [7,13,14], yet little work has been done to understand how transformers model physics. This question is crucial, as for transformers to build any sort of “world model” they must have a grasp of the physical laws that govern the world [15].

**Figure 1:**
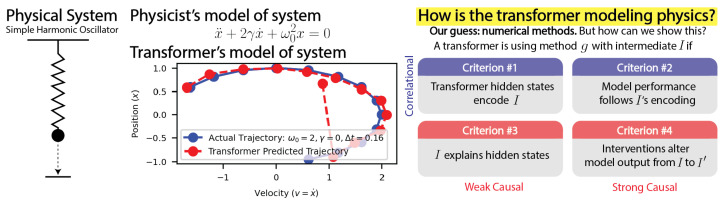
We aimed to understand how transformers model physics through the study of meaningful intermediates. We trained transformers to model simple harmonic oscillator (SHO) trajectories, and we used our developed criteria of intermediates to show that transformers use known numerical methods to model the SHO.

Our key research question was the following: How do transformers model physics? This question is intimidating, since even humans have many different ways of modeling the same underlying physics [16]. In the spirit of hypothesis testing, we reformulated the question as follows: Given a known modeling method *g*, does the transformer learn *g*? If a transformer leverages *g*, its hidden states must encode information about important intermediate quantities in *g*. We focused our study on the simple harmonic oscillator $\ddot{x}+2\gamma \dot{x}+\omega_{0}^{2}x=0$, where γ and $\omega_{0}$ are the damping and frequency of the system, respectively. Given the trajectory points $\left\{\left(x_{0},v_{0}\right),\left(x_{1},v_{1}\right),\ldots ,\left(x_{n},v_{n}\right)\right\}$ at discrete times $\left\{t_{0},t_{1},\ldots ,t_{n}\right\}$, we tasked a transformer with predicting $\left(x_{n+1},v_{n+1}\right)$ at time $t_{n+1}$, as shown in Figure 1. In this setting, *g* could be a numerical simulation the transformer runs after inferring $\gamma ,\omega_{0}$ from past data points. We would then expect some form of γ and $\omega_{0}$ to be intermediates encoded in the transformer. How, we asked, could we show that intermediates and the method *g* were being used?

We developed criteria to demonstrate that the transformer was using *g* by studying intermediates in a simpler setting: in-context linear regression, y=wx. As correlational evidence for the model’s internal use of *w*, we found that the intermediate *w* can be encoded linearly, nonlinearly, or not at all. We also linked the performance of models to their encoding of *w*, and we used this as an explanation for in-context learning. We generated causal evidence for the use of *w* by analyzing how much of the hidden states’ variance was explained by *w* and by linearly intervening in the network, to predictably change its behavior.

We used these developed criteria of intermediates to study how transformers model the simple harmonic oscillator (SHO), a fundamental model in physics. We generated multiple hypotheses for the method(s) that transformers use to model the trajectories of SHOs, and we used our criteria from linear regression to show correlational and causal evidence that transformers employ known numerical methods, specifically the matrix exponential, to model trajectories of SHOs. Although our analysis was constrained to the SHO in this paper, our framework naturally extends to some high-dimensional linear and nonlinear systems.

The organization of this paper is as follows. In Section 2, we overview related work. In Section 3, we define and investigate intermediates in the setting of linear regression, and we use this to develop criteria for transformers’ use of a method *g*. In Section 4, we hypothesize that transformers use numerical methods to model the SHO, and we use our criteria of intermediates to provide causal and correlational evidence for transformers’ use of the matrix exponential.

## 2. Related Work

**Mechanistic interpretability.** Mechanistic interpretability (MI), as a field, aims to understand the specific computational procedures machine learning models use to process inputs and produce outputs [5,6,7,8,9,10,11,12,13]. Some MI work focuses on decoding the purpose of individual neurons [17], while other work focuses on ensembles of neurons [11,12]. Our work is aligned with the latter.

**Algorithmic behaviors in networks.** A subset of MI attempts to discover the specific algorithms that networks use to solve tasks by reverse engineering weights. For example, it has been demonstrated that transformers use the discrete Fourier transform to model modular addition [13]. Reverse engineering has been studied extensively for neural circuits, which has, impressively, connected the behavior of neurons and brain areas [18,19]. We focused on transformers, and, instead of reverse engineering weights, we made use of linear probing [20] to discover byproducts of algorithms represented internally by transformers. Studies have found that algorithms in models are potentially an “emergent” behavior that manifests with size [21,22], which we also found.

**AI and Physics.** Many works design specialized machine learning architectures for physics tasks [23,24,25,26,27], but less work has been undertaken to see how well transformers perform on physical data out of the box. Recently, it was shown that LLMs can in-context learn physics data [15], which inspired the research question of this paper: how do transformers model physics?

## 3. Developing Criteria for Intermediates with Linear Regression

Our main goal was to determine which methods transformers use to model the simple harmonic oscillator. We aimed to do this by generating criteria based on the encoding of relevant intermediates. For this section, we developed our criteria of intermediates in a simpler setting: linear regression. Notably, linear regression is identical to predicting the acceleration from the position of an undamped harmonic oscillator (γ=0), making this setup physically relevant.

**Setup.** In our linear regression setup, we generated X and w between [−0.75,0.75], where X had size (5000,65) and w had size (5000). We generated Y=wX, and we trained the transformers to predict $y_{n+1}$ given $\left\{x_{1},y_{1},...,x_{n},y_{n},x_{n+1}\right\}$.

Since in-context linear regression is well studied for transformers [28,29], we used this simple setting to ask and answer fundamental questions about intermediates, namely:

- **What** is an intermediate?
- **How** can intermediates be encoded and how can we robustly probe for them?
- **When**, or under what circumstances, are intermediates encoded?

All of these questions developed an understanding of intermediates that built up to the **key question: How can we use intermediates to demonstrate that a transformer is actually using a method in its computations?** By answering this question for linear regression, we generated four correlational and causal criteria to demonstrate that a transformer is using a method in its computations, which we could then apply to understanding the simple harmonic oscillator, as shown in Figure 1.

### 3.1. What Is an Intermediate?

We define an intermediate as a quantity that a transformer uses during computation, but which is not a direct input/output to/of the transformer. More formally, if the input to the transformer is X and its output is Y, we can model the transformer’s computation as Y=g(X,I), where *g* is the method used and *I* is the intermediate of that method. For example, if we want to determine if the transformer is computing the linear regression task using Y=wX then I=w, g(X,I)=g(X,w)=wX.

### 3.2. How Can Intermediates Be Encoded and How Can We Robustly Probe for Them?

We wanted to understand what form of the intermediate, f(I), is encoded in the network’s hidden states. For example, while it may be obvious to humans to compute y=wx, perhaps transformers prefer exp(log(w)+log(x)) or $\sqrt{w^{2}x^{2}}$. We wanted to develop a robust probing methodology that captures these diverse possibilities. We identified three ways an intermediate *I* can be represented: linearly encoded, nonlinearly encoded, and not encoded at all. We used HS to mean hidden state.

**Linearly encoded.** We say *I* is linearly encoded in a hidden state HS if there is a linear network that takes I=Linear(HS). We determine the strength of the linear encoding by evaluating how much of the variance in *I* can be explained by HS, i.e., the $R^{2}$ of the probe.

**Nonlinearly encoded.** To probe for an arbitrary f(I), we define a novel **Taylor probe**, which finds coefficients $a_{i}$, such that $f\left(I\right)=a_{1}I+a_{2}I^{2}+...+a_{n}I^{n}$, and f(I)=Linear(HS).

To actually implement this probing style, we use Canonical Correlation Analysis probes, which, given some multivariate data *X* and *Y*, find directions within *X* and *Y* that are maximally correlated [30]. Here, $X=[I,I^{2},I^{3},...,I^{n}]$ and Y=HS. If *I* is of bounded magnitude and *n* is sufficiently large, we are able to probe the transformer for any function f(I). In practice, we used n≤5.

**Not encoded.** If *I* fails to be linearly or nonlinearly encoded, we say that it is not encoded within the network. For example, there are at least two ways to predict $y_{2}$ from $\left\{x_{1},y_{1},x_{2}\right\}$, such that $y_{2}=\frac{y_{1}}{x_{1}}x_{2}$: (1) $w=y_{1}/x_{1}$ is encoded, and $y_{2}=wx_{2}$; (2) $w^{\prime }=x_{2}/x_{1}$ is encoded (so $w=y_{1}/x_{1}$ is not encoded) and $y_{2}=w^{\prime }y_{1}$. Thus, it is not guaranteed that *w* is encoded.

### 3.3. When, or Under What Circumstances, Are Intermediates Encoded?

**Figure A1:**
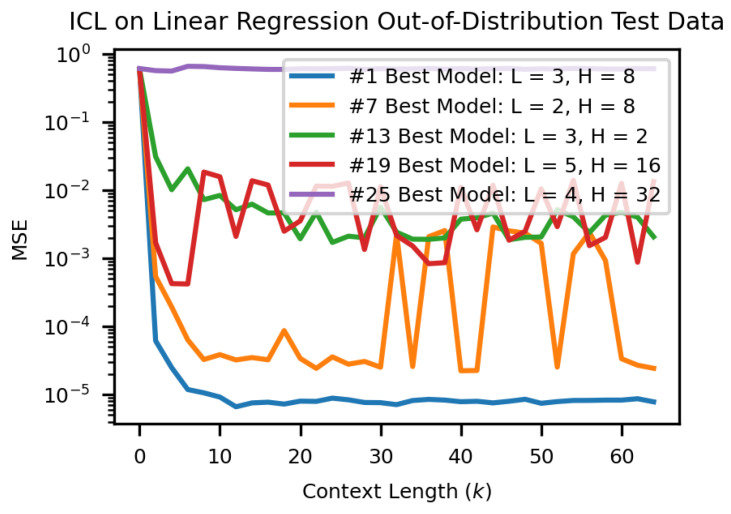
We found that the linear regression models were able to generalize to out-of-distribution test data with 0.75≤|w|≤1.

We wanted to apply our probing techniques to better understanding what type of models generate intermediates. Under the described setting of linear regression, we trained GPT style transformers of size L=[1,2,3,4,5] and H=[2,4,8,16,32], where *L* was the number of layers and *H* was the hidden size of the transformer. All the transformers trained in this study used one attention head and no LayerNorm to aid interpretability, and they were trained on a NVIDIA Volta GPU with the hyperparameters $\mathrm{epochs}=20,000,\;\mathrm{lr}=10^{-3},\;\mathrm{batchsize}=64$, using the Adam optimizer [31]. We did not use a token embedding—instead, the inputs were raw numbers representing the position of the SHO. We found that these models generalized to the out-of-distribution test data (0.75≤|w|≤1) in Appendix A Figure A1, but we focused on investigating the intermediates on in-distribution training data.

**Figure 2:**
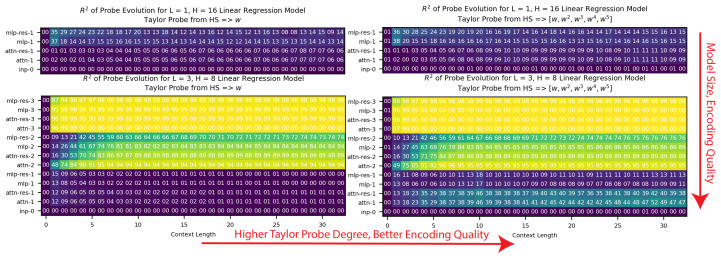
We plotted the $R^{2}$ of the Taylor probes for the intermediate w within the models trained on the task Y=wX (linear regression). We saw that the larger models had w encoded, often linearly, with little gain as we moved to higher-degree Taylor probes, while the smaller models did not have w encoded.

**Figure A2:**
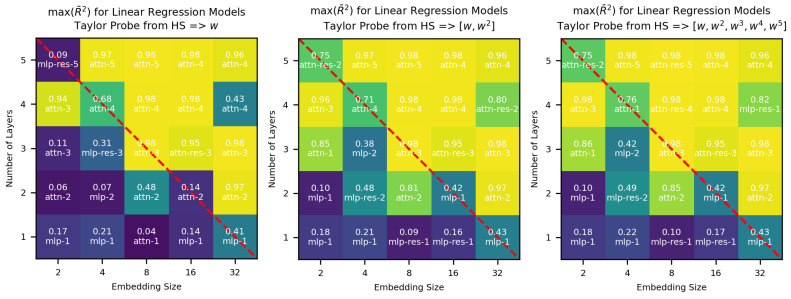
We calculated the mean of the $R^{2}$ of probes for f(w) across all layers of the transformer and we annotated each model with its highest mean score, $\max(\bar{R^{2}})$. When f(w) was linear (**left**) and quadratic (**middle**), we observed a striking phase transition of encoding based on model size, demarked by the red dashed line. If w was encoded, it was mostly encoded linearly, with the (L,H)=(5,2),(4,32),(2,8) models showing signs of a quadratic representation of w. We did not see any meaningful gain in encoding when extending the Taylor probe to degree n>2 (**right**). For the models where f(w) was well represented, it often happened in the attention layer. This was possibly because the attention layer aggregated all past estimates of f(w) into an updated estimate.

**Larger models have stronger encodings of intermediates.** We found that the smaller models often did not have w encoded, while the larger models encoded w linearly, as evidenced by Figure 2. We formalized this further by defining $\max(\bar{R^{2}})$ as the maximum value taken over the depth positions of the mean $R^{2}$ of the w probes taken over the context length. As shown in Appendix A Figure A2, we observed a clear phase transition in encoding across model size, and we also found that $\max(\bar{R^{2}})$ did not significantly improve if we extended the degree of the Taylor probes to n>2. Thus, in the case of linear regression, we found that the models represented w linearly, quadratically, or not at all.

We attributed the stronger encoding of w in the larger models to the “lottery ticket hypothesis”—larger models have more “lottery tickets” in their increased capacity to find a “winning” representation of w [32,33]. Interestingly, the intuitive understanding that larger models have w better encoded led us to the counterintuitive conclusion that larger models are actually more interpretable for our purposes.

**Figure 3:**
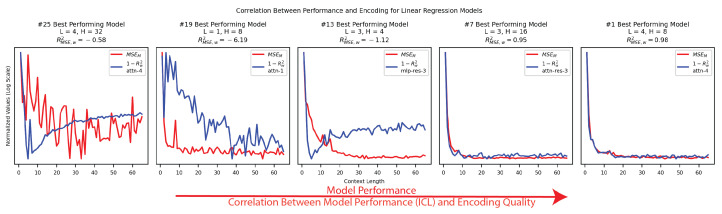
We tested the correlation between model performance and the encoding of w on 5 of our 25 linear regression models of evenly spaced performance quality. We plotted normalized values for the error of the encoding ($1-R_{w}^{2}$) in red and the mean squared error of the model (${MSE}_{M}$) in blue. We found that the ability of the best-performing models to in-context learn was highly correlated with their encoding of w ($R^{2}\left(MSE,w\right)$.

**Figure A3:**
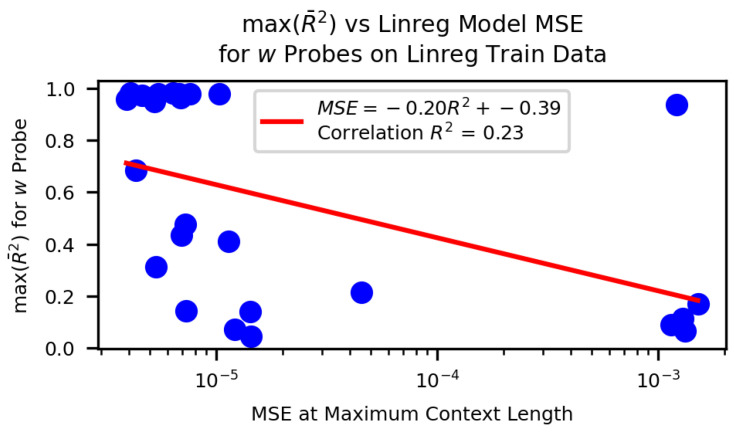
Better-performing models generally had better encodings of w, while worse-performing models generally had worse encodings (other than one outlier in the top-right).

**Encoding quality is tied to model performance.** As shown in Appendix A Figure A3, we found that the better-performing models generally had stronger encodings of w. As shown in Figure 3, we also found that the improvements in model prediction as a function of context length, or in-context learning, were correlated to improvements in w’s encoding, which we would expect if our models were using w in their computations.

### 3.4. Key Question: How Can We Use Intermediates to Demonstrate That a Transformer Is Actually Using a Method in Its Computations?

So far, we had discovered that models encode w, either linearly or nonlinearly, and we had found relationships between model size, performance, and encoding strength. But how, we asked, could we ensure that the model was actually using w in its computations and that the encoding of w was not just a meaningless byproduct [34]?

**Figure 4:**
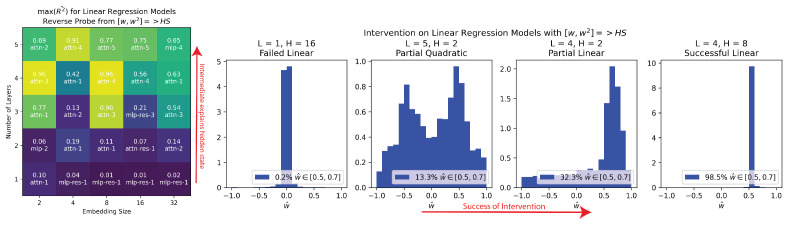
Left: We plotted $\max(\bar{R^{2}})$ of the reverse probe from $[w,w^{2}]\to HS$ across all the linear regression models, and we found that the intermediate w could explain significant amounts of variance in the model hidden states. Right: We intervened, using reverse probes to make all the models output $w^{\prime }=0.5$. This intervention failed (16/25), it was partially successful nonlinearly (2/25) or linearly (3/25), or it was successful (4/25). We noted the empirically observed w as $\hat{w}$ calculated by $\hat{y}/x$ where $\hat{y}$ was the output of the intervened transformer and *x* was the input.

**Reverse Probing.** To ensure that w was not encoded in some insignificant part of the residual stream, we set up probes going from $[w,w^{2}]\to HS$, as opposed to HS→f(w). As shown in Figure 4, we often found that w could explain large amounts of variance in model hidden states, implying that these hidden states were dedicated to representing w. We took this as weak causal evidence that w was being used by the model—otherwise, it was unclear why a part of the model would be dedicated to storing w.

**Intervening.** We could also use reverse probes to intervene in the models’ hidden states and predictably change their output from $w\to w^{\prime }$. As shown in Figure 4, we attempted to make $w^{\prime }=0.5$ for all series and then measure the observed $\hat{w}$ from the models’ outputs ($\hat{w}={\hat{y}}_{n}/x_{n}$). For 4 out of 25 models the intervention worked, providing strong causal evidence that the model uses its internal representation of w in computations. For models where we identified a quadratic representation of w, we see that w=0.5,−0.5 were both represented in the observed intervention.

**Putting it all together.** We were able to generalize our understanding of intermediates from linear regression, to create criteria for a transformer’s use of a method *g* in its computations.

**Criteria for use of a method** *g* **with an associated unique intermediate** *I***:**

1. If a model uses a method *g* then its hidden states should encode *I* (shown in Figure 2).
2. If a model uses a method *g* then the model performance should improve if *I* is better represented (shown in Figure 3).
3. If and only if the model uses *g* then we expect some hidden state’s variance to be almost fully explained by *I* (shown in Figure 4).
4. If and only if the model uses *g* then we can intervene with hidden states, to change $I\to I^{\prime }$ and predictably change the model output from $g\left(X,I\right)\to g\left(X,I^{\prime }\right)$ (shown in Figure 4).

The first two criteria for a transformer’s use of *g* are correlational, and the last two are weak and strong causal. Using these criteria (summarized in Figure 1), we could then investigate how transformers model more complex systems like the simple harmonic oscillator.

## 4. Investigating the Simple Harmonic Oscillator

We next applied our developed criteria of intermediates to investigating how transformers represent physics, specifically the methods they use to model the simple harmonic oscillator (SHO). The simple harmonic oscillator is ubiquitous in physics: it is used to describe phenomena as diverse as the swing of a pendulum, molecular vibrations, the behavior of AC circuits, and quantum states of trapped particles. Given a series of position and velocity data for a simple harmonic oscillator at a sequence of timesteps, we asked

1. Can a transformer successfully predict the position/velocity at the SHO’s next timestep?
2. Can we determine what computational method the transformer is using in this prediction?

### 4.1. Mathematical and Computational Setup

The simple harmonic oscillator is governed by the linear ordinary differential equation (ODE):

(1) $$\ddot{x}+2\gamma \dot{x}+\omega_{0}^{2}x=0.$$

**Figure A4:**
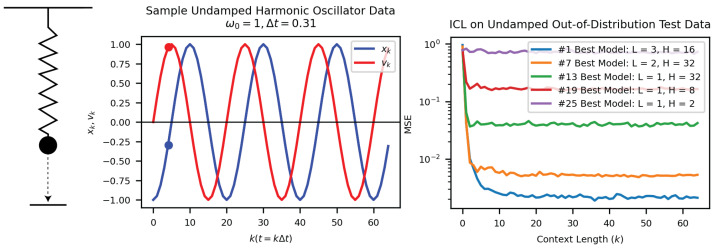
An intuitive picture for a simple harmonic oscillator is a mass oscillating on a spring (**left**). The trajectory of the SHO can be fully parameterized by the value of x,v at various timesteps (**middle**), and we found that models trained to predict undamped SHO trajectories are able to generalize to out-of-distribution test data with in-context examples (**right**).

The two physical parameters of this equation are γ, the damping coefficient, and $\omega_{0}$, the natural frequency of the system. An intuitive picture for the SHO is a mass on a spring that is pulled from its equilibrium position by some amount $x_{0}$ and let go, as visualized in Figure 1; $\omega_{0}$ is related to how fast the system oscillates, and γ is related to how soon the system decays to equilibrium from the internal resistance of the spring. We focused on studying how a transformer modeled the undamped harmonic oscillator, where γ=0. Given some initial starting position ($x_{0}$), velocity ($v_{0}$), and timestep Δt, the time evolution of the undamped harmonic oscillator was

(2) $$\begin{array}{cc} x_{k} & =x_{0}\cos\left(k\omega_{0}\Delta t\right)+\frac{v_{0}}{\omega_{0}}\sin\left(k\omega_{0}\Delta t\right)\\ v_{k} & =v_{0}\cos\left(k\omega_{0}\Delta t\right)-\omega_{0}x_{0}\sin\left(k\omega_{0}\Delta t\right), \end{array}$$

where $v=\frac{dx}{dt}$. We generated 5000 timeseries of 65 timesteps for various values of $\omega_{0},\Delta t,x_{0},$ and $v_{0}$, described in Appendix C. Following the procedure for linear regression, we trained transformers of size L=[1,2,3,4,5] and H=[2,4,8,16,32] to predict $\left(x_{n+1},v_{n+1}\right)$, given $\left\{\left(x_{0},v_{0}\right),\left(x_{1},v_{1}\right),\ldots \left(x_{n},v_{n}\right)\right\}$. In Appendix C Figure A4, we see that our transformers were able to accurately predict the next timestep in the timeseries of out-of-distribution test data, and this prediction became more accurate with context length (i.e., in-context learning). But how was the transformer modeling the simple harmonic oscillator internally?

### 4.2. What Methods Could the Transformer Use to Model the Simple Harmonic Oscillator?

Human physicists would model the simple harmonic oscillator with the analytical solution to Equation (1), but it is unlikely that a transformer does so. Transformers are numerical approximators that use statistical patterns in data to make predictions, and, in that spirit, we hypothesize that transformers use numerical methods to model SHOs. There is a rich literature on numerical methods that approximate solutions to linear ordinary differential equations [35,36,37], and we highlight three possible methods the transformer could be using in our theory hub. For notation, we note that Equation (1) can be written as

(3) $$\left[\begin{array}{c} \dot{x}\\ \dot{v} \end{array}\right]=\left[\begin{array}{cc} 0 & 1\\ -\omega_{0}^{2} & -2\gamma \end{array}\right]\left[\begin{array}{c} x\\ v \end{array}\right]=A\left[\begin{array}{c} x\\ v \end{array}\right].$$

**Linear Multistep Method.** Our model could have been using a linear multistep method, which uses values of derivatives from several previous timesteps to estimate the future timestep. We describe the *k*th order linear multistep method in Table 1 with coefficients $\alpha_{j}$ and $\beta_{j}$.

**Taylor Expansion Method.** The model could also have been using higher-order derivatives from the previous timestep to predict the next timestep (this is equivalent to the nonlinear single-step Runge–Kutta method for a homogeneous linear ODE with constant coefficients). We describe the *k*th order Taylor expansion in Table 1.

**Matrix Exponential Method.** While the two methods presented above are useful approximations for small Δt, the matrix exponential uses a 2×2 matrix to exactly transform the previous timestep to the next timestep. We describe this in Table 1. This method is the $\lim_{k\to \infty }$ of the Taylor expansion method.

In order to use the criteria described in Section 3 to figure out which method(s) our model was using, we needed to define the relevant intermediates for each method *g*. Similarly to the linear regression, the intermediates were the coefficients of the input, but were now 2×2 matrices and not a single value. We summarize our methods and intermediates in our theory hub in Table 1. Notably, these methods are viable for any homogeneous linear ordinary differential equation with constant coefficients and potentially for nonlinear differential equations as well (see Appendix B).

### 4.3. Evaluating Methods for the Undamped Harmonic Oscillator

**Figure A6:**
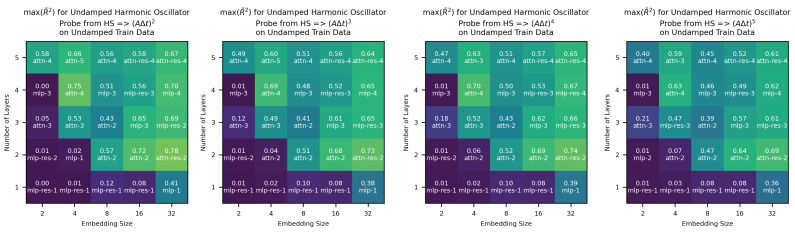
We found that our choice of *j* in the intermediate for the Taylor expansion method (${\left(A\Delta t\right)}^{j}$) had little effect on our results or conclusions about the undamped harmonic oscillator (shown for criterion 1).

We applied the four criteria established for linear regression (Figure 1) to evaluating if transformers use the methods in Table 1. For the Taylor expansion intermediate, we used j=3 to distinguish it from the linear multistep method, although our results were generally robust for j≤5 (Appendix C Figure A6). We summarize our evaluations across the methods and criteria in Table 2.

**Figure 5:**
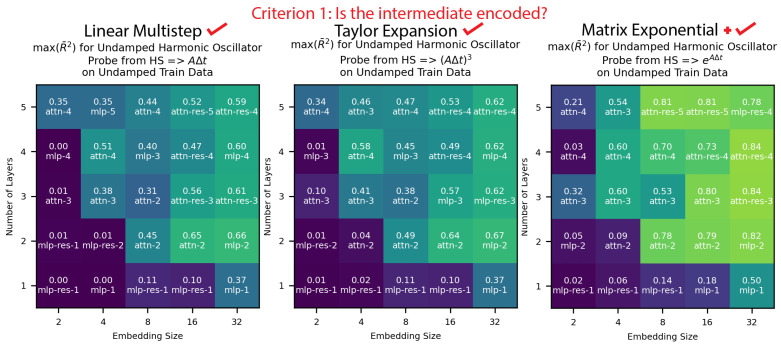
We analyzed the intermediates of our undamped harmonic oscillator models, and we found all three methods encoded, with the matrix exponential method best represented. This provided initial correlational evidence for all three methods.

**Figure 6:**
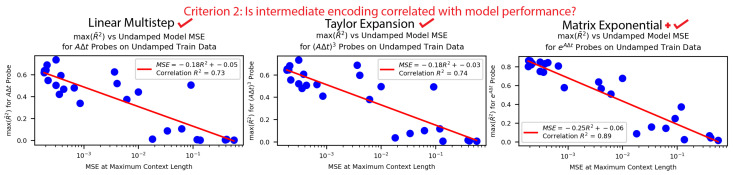
We found that the better-performing undamped harmonic oscillator models had intermediates of all methods better encoded, but this correlation was strongest in magnitude and slope for the matrix exponential method. This was additional correlational evidence for all three methods.

**Criterion 1: Is the intermediate encoded?** In Figure 5, we see that all three intermediates were well encoded in the model, with the matrix exponential method especially prominent. This provides initial correlational evidence that the models were learning numerical methods. The magnitude of the encodings was generally smaller than the linear regression case, which we attribute to the increased difficulty of encoding 2×2 matrices compared to a single weight value w. Notably, we only probed for linear encodings, given that w was most often encoded linearly in the linear regression case study:

**Criterion 2: Is the intermediate encoding correlated with the model performance?** In Figure 6, we see that for all three methods the better-performing models generally had stronger encodings and the worse-performing models had weaker encodings. This correlation was strongest for the matrix exponential method. This provided more correlational evidence that our models were using the described methods.

**Figure 7:**
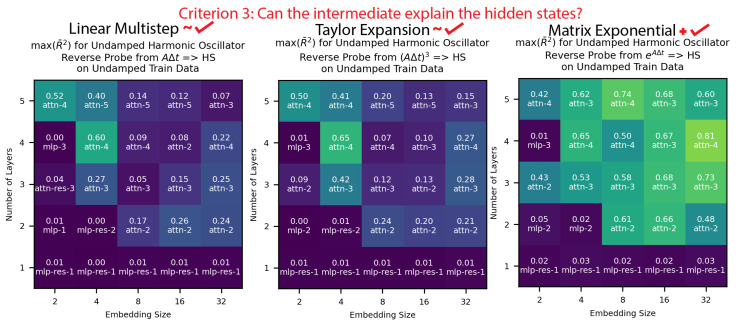
We found that the intermediates from all three methods could explain some variance in the undamped harmonic oscillator model hidden states, but that the matrix exponential method was the most consistent and successful by a wide margin.

**Criterion 3: Can the intermediates explain the models’ hidden states?** As shown in Figure 7, we reverse probed from the intermediates to the models’ hidden states, and we found that all the methods explained non-trivial variance in the model hidden states, while the matrix exponential method consistently explained the most variance by a sizable margin. This provided a little weak causal evidence that the models were using the linear multistep and Taylor expansion methods and stronger weak causal evidence that the models were using the matrix exponential method.

**Figure 8:**
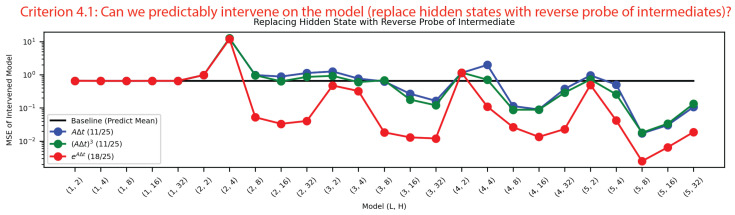
For each undamped harmonic oscillator model and method, we replaced the hidden state in Figure 7 with the reverse probe of the intermediate. We can see that this intervention was consistently the best performing for the matrix exponential method by an order of magnitude, and that 18/25 models performed better than our baseline of guessing.

**Criterion 4: Can we predictably intervene on the the model?** Criterion 4.1: To intervene on the model, we used the reverse probes from Figure 7 to generate predicted hidden states from each intermediate. As shown in Figure 8, we then inserted these hidden states back into the model, to see if the model was still able to model the SHO. The matrix exponential method had the most successful interventions by an order of magnitude, and 18/25 of these intervened models performed better than guessing. This implies that the information the transformer uses to model the SHO is stored in the matrix exponential’s intermediate.

**Figure 9:**
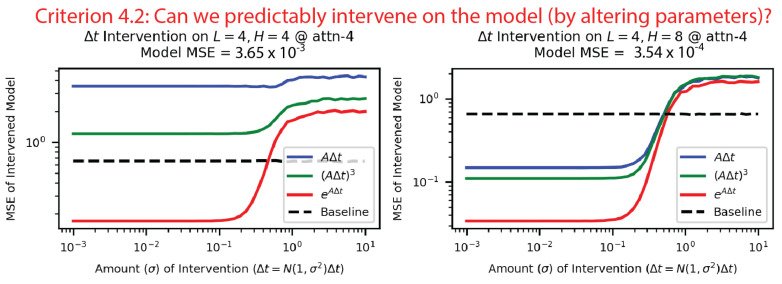
We varied the value of Δt used in the intermediates, and we used the reverse probes from Figure 7 to generate hidden states from these intermediates. We performed this operation on two undamped harmonic oscillator models, which had the best linear multistep/Taylor expansion (L=4,H=4) and matrix exponential (L=4,H=8) reverse probes, respectively, and we found that the matrix exponential was consistently most robust for interventions. The baseline was if our model only predicted the mean of the dataset.

**Figure A7:**
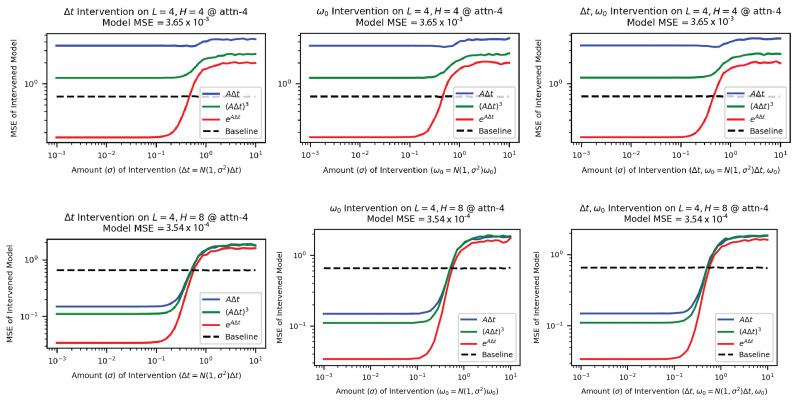
Regardless of which quantities we intervened on, our general results were robust for criterion 4 for the undamped harmonic oscillator.

Criterion 4.2: We could also vary $\Delta t\to \Delta t^{\prime },\omega_{0}\to \omega_{0}^{\prime }$, regenerate intermediates and then hidden states, insert these modified hidden states into the model, and see if the model made predictions as if it “believed” that the input SHO data used $\Delta t^{\prime },\omega^{\prime }$. As shown in Figure 9, we performed this intervention on Δt, but our results were sufficiently robust to intervene on $\omega_{0}$ as well (Appendix C Figure A7). Even for the model with the best reverse probe quality for the linear multistep/Taylor expansion intermediates (L=4,H=4), the intervention with the matrix exponential method was the most successful. Combined with our previous intervention (4.1), we now had strong causal evidence for the matrix exponential method.

**Figure A8:**
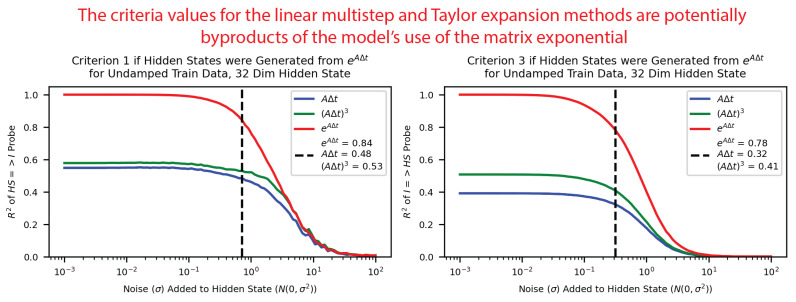
We generated synthetic hidden states from the matrix exponential intermediates and found that this naturally resulted in values for criterion 1,3 for the linear multistep and Taylor expansion methods that were close to those we observe in Table 2. This is correlational evidence that the matrix exponential method was potentially solely used by the transformer, and that the values for the other two methods were byproducts. These byproducts could arise because $e^{A\Delta t}=\sum_{j}{\left(A\Delta t\right)}^{j}/j$.

**The transformer likely uses the matrix exponential to model the undamped harmonic oscillator.** We now had correlational evidence that the model was using all three methods in our theory hub, with little causal evidence for the linear multistep and Taylor expansion methods, and strong causal evidence for the matrix exponential method. We suspected the model was only using the matrix exponential method in its computations, and that the evidence we had for the other two methods was a byproduct of the use of the matrix exponential. In Appendix C Figure A8, we give correlational evidence for this claim by generating synthetic hidden states from $e^{A\Delta t}$ and showing that in this synthetic setup we retrieved values for criterions 1, 3 for linear multistep and Taylor expansion that were close to those we observed in Table 2.

**Table 2:** We summarize the evaluation of the methods and criteria for the undamped/underdamped models. For each criterion, we list a single quantity for readability: criterion 1 is the largest value in Figure 5, criterion 2 is the correlation in Figure 6, criterion 3 is the largest value in Figure 7, and criterion 4 is the ratio in the legend of Figure 8. We bold the best performing hypothesis for each criteria. The matrix exponential performed best across the criteria.

Criterion	Linear Multistep	Taylor Expansion	Matrix Exponential
1. Intermediate encoding	0.66/0.51	0.67/0.25	**0.84**/**0.54**
2. Performance, encoding correlation	0.73/**0.44**	0.74/0.39	**0.89**/**0.44**
3. Intermediate’s explanatory power	0.42/0.15	0.53/0.11	**0.78**/**0.16**
4. Intervention success	0.44/*X*	0.44/*X*	**0.72**/*X*

Thus, we concluded that the transformer was likely using the matrix exponential method. This made sense, given the problem setting—both the linear multistep and Taylor expansion methods were only accurate for small Δt, while our bound of $\Delta t=U[0,2\pi /\omega_{0}]$ violated this assumption for some timeseries. On the other hand, the matrix exponential makes no such assumptions for the timestep, and it is, thus, a more general method that the transformer can employ to flexibly model a variety of situations. Still, it is remarkable that transformers use a known numerical method to model the undamped harmonic oscillator, and that we can provide evidence for its use, although our experiments do not rule out the possibility of other methods being used in conjunction with the matrix exponential.

### 4.4. Extension to the Damped Harmonic Oscillator (γ≠0)

We wanted to understand the generality of our finding by extending our problem space to the damped harmonic oscillator, where γ≠0. We have left the relevant details about our procedure to Appendix D, but, as shown in Table 2, we found that our intermediate analysis performed much more poorly on the underdamped case than on the undamped. We describe possible explanations in Appendix D, but because of this we temper our finding from the undamped harmonic oscillator with caution about its generality.

## 5. Discussion

After developing criteria for intermediates in the toy setting of linear regression, we found that transformers use known numerical methods for modeling the simple harmonic oscillator, specifically the matrix exponential method. We leave the door open for researchers to better understand the methods transformers use to model the damped harmonic oscillator and to use the study of intermediates to understand how transformers model other systems in physics.

**Limitations.** We analyzed relatively small transformers with only one attention head and no LayerNorm. While we demonstrated strong results for the undamped harmonic oscillator, our results for the underdamped harmonic oscillator were more mild. We only used noiseless data.

## Figures and Tables

**Table 1:** Our theory hub of numerical methods and relevant intermediates that transformers could be using to model the simple harmonic oscillator.

Method	g(X,I)	*I*
Linear Multistep	$\left[\begin{array}{c} x_{n+1}\\ v_{n+1} \end{array}\right]=\sum_{j=0}^{k}\alpha_{j}\left[\begin{array}{c} x_{n-j}\\ v_{n-j} \end{array}\right]+\sum_{j=-1}^{k}\beta_{j}A\Delta t\left[\begin{array}{c} x_{n-j}\\ v_{n-j} \end{array}\right]$	AΔt
Taylor Expansion	$\left[\begin{array}{c} x_{n+1}\\ v_{n+1} \end{array}\right]=\sum_{j=0}^{k}A^{j}\frac{\Delta t^{j}}{j!}\left[\begin{array}{c} x_{n}\\ v_{n} \end{array}\right]$	${\left(A\Delta t\right)}^{j}$
Matrix Exponential	$\left[\begin{array}{c} x_{n+1}\\ v_{n+1} \end{array}\right]=e^{A\Delta t}\left[\begin{array}{c} x_{n}\\ v_{n} \end{array}\right]$	$e^{A\Delta t}$

**Figure A5:**
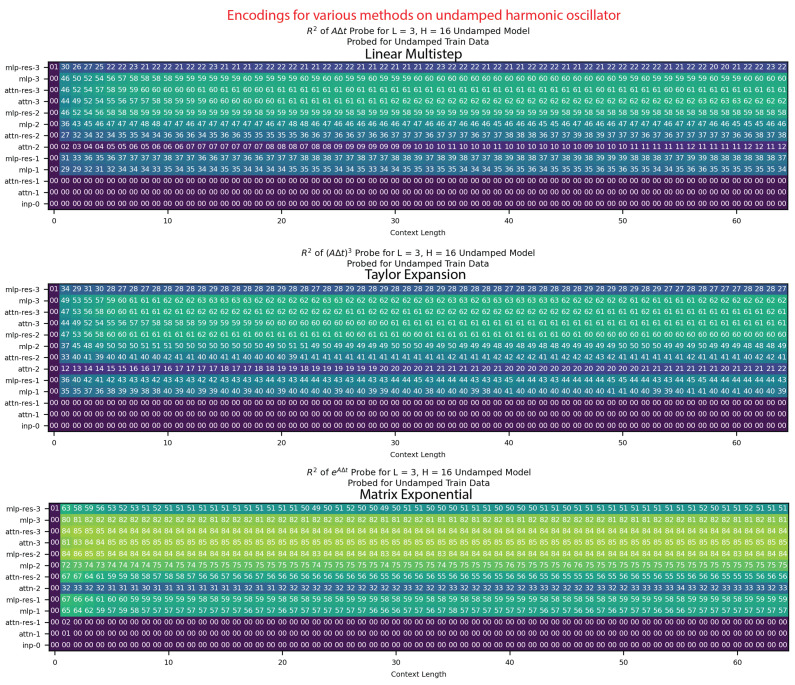
We visualize the evolution of encodings across all the methods, with context length for the best-performing undamped model.

**Figure A9:**
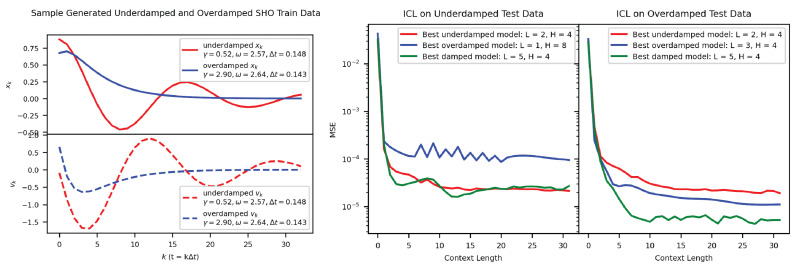
We generated data for the underdamped and overdamped harmonic oscillators following the procedure detailed in Section 3, and we visualize the sample curves in the left-most plot. From both the analytical equations and the plotted curves, we see that the underdamped and the overdamped data followed very different trajectories. Amazingly, on the right-most plot we find that the transformers trained on the underdamped data generalized to overdamped data. This implies that our transformer was using a similar method to calculate both, otherwise this generalization would be impossible. We hypothesize that our “AI Physicist” was using one of the numerical methods from the undamped case. Note that the “damped” oscillator was trained on equal parts underdamped and overdamped data.

**Figure A10:**
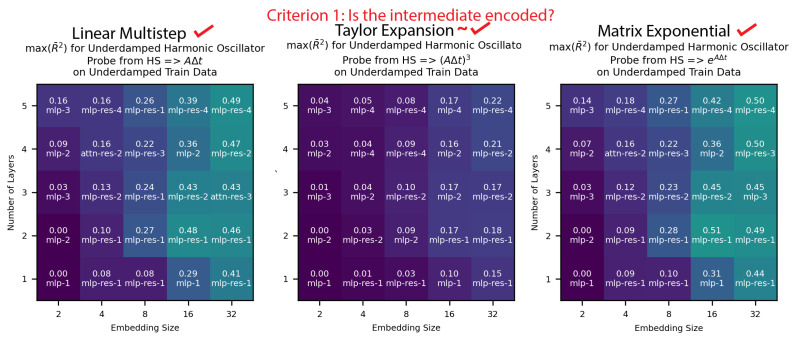
We observe that the intermediates for all three methods were encoded, but they were less than the undamped case in Figure 5. The linear multistep was roughly as prominent as the matrix exponential method, which was also a departure from the undamped case.

**Figure A11:**
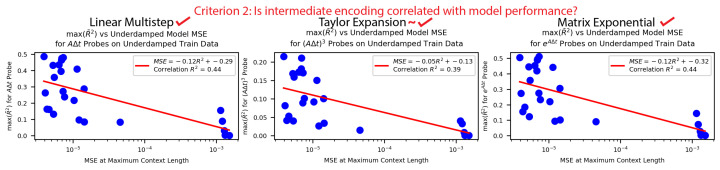
We see that, generally, the better-performing models exhibited stronger encodings of intermediates, while the worse-performing models exhibited weaker encodings. These trends were not as strong as the undamped case, shown in Figure 6. Like criterion 1 in Figure A10, we see that the linear multistep method was competitive with the matrix exponential method.

**Figure A12:**
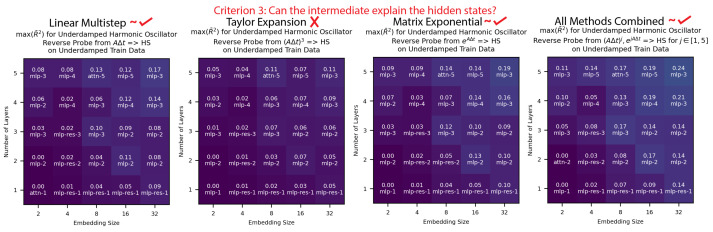
Multiple methods represented nontrivial amounts of variance in the hidden states, but even all the methods combined (right) explained less than a quarter of the variance in the hidden states.

**Figure A13:**
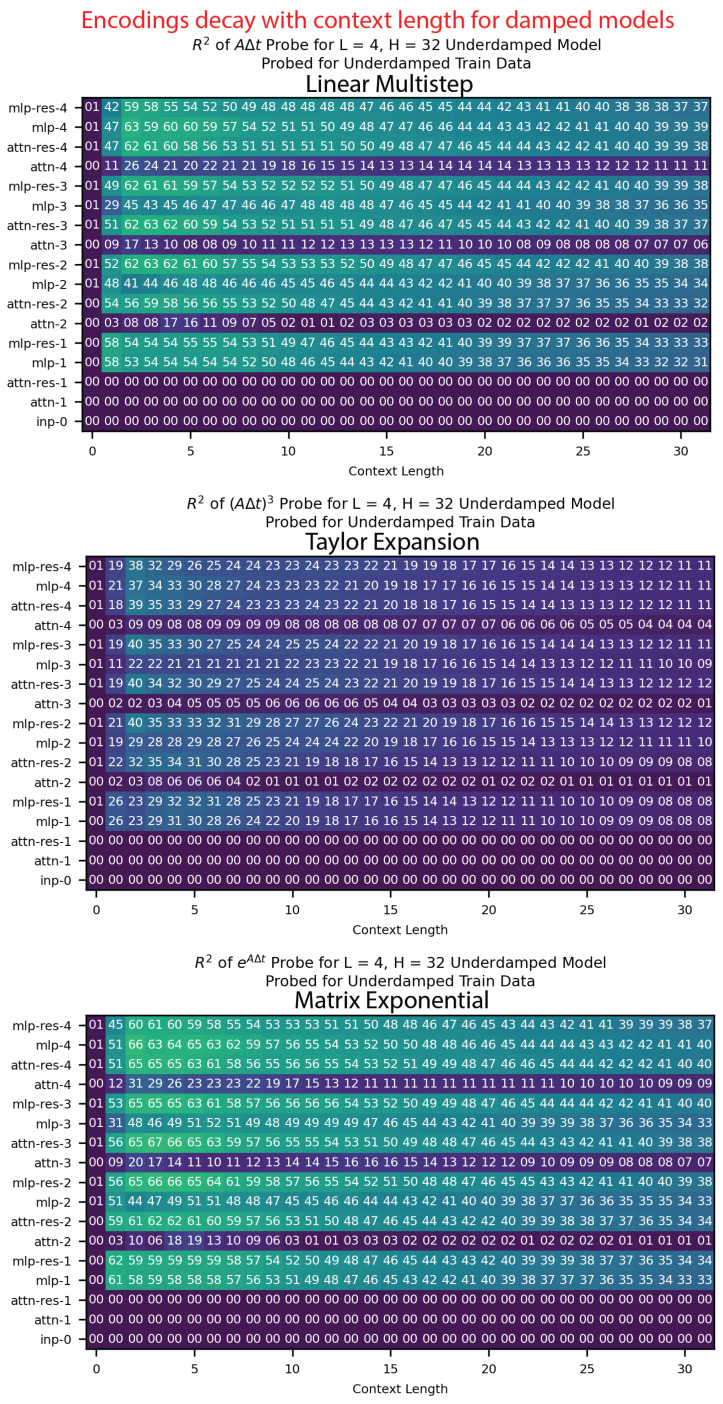
We see that the encoding strength of the intermediates decayed across all the methods with context length. This similarly matched the natural decay to 0 of the damped harmonic oscillator, and it is one potential explanation for why our methods were not as prominent in the damped vs. undamped cases, for which the encoding quality did not decay with context length (Figure A13). While this is a general observation across the models, we visualize the L=4, H=32 model because it had the strongest encoding of intermediates from Figure A10.

**Figure A14:**
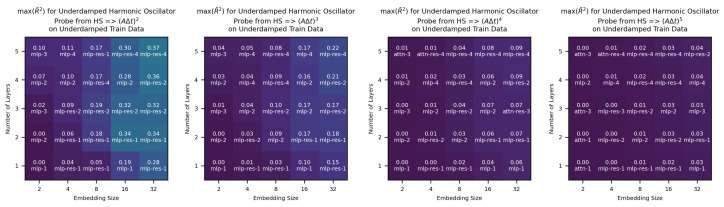
We found that our choice of *j* in the intermediate for the Taylor expansion method (${\left(A\Delta t\right)}^{j}$ had a major effect on the encoding quality, unlike the undamped case visualized in Figure A6. We see that j>3 was very poorly represented in the transformer, which implies that if the transformer was using the Taylor expansion for the underdamped spring, it would likely be of order k=3 or less.

## Data Availability

Relevant code can be found at https://github.com/subhashk01/transformer-physics (accessed on 6 November 2024). This includes the scripts that generated all the data used in this study. Contact subhashk@mit.edu directly for more information.

## Appendix A. Additional Results for Linear Regression

As shown in Figure A1, we found that our transformers were able to generalize to linear regression test examples with out-of-distribution data (0.75≤|w|≤1). In Figure A2, we see that the smaller models did not have w encoded, while the larger models often had w linearly encoded (with some quadratic encodings as well). In Figure A3, we see that the better-performing models generally had better encodings, while the worse-performing models generally had worse encodings.

## Appendix B. Undamped Harmonic Oscillator Appendices

We note that the theory hub we summarized in Table 1 is valid for all differential equations that can be written as $\dot{x}=\mathrm{Ax}$ if A is a constant matrix. This includes all homogeneous linear differential equations with constant coefficients and potentially nonlinear differential equations as well. The Koopman operator theory allows nonlinear differential equations to be modeled as linear differential equations. Here is an example taken from [39]:

Here, we consider an example system with a single fixed point, given by:

(A1a) $${\dot{x}}_{1}=\mu x_{1}$$

(A1b) $${\dot{x}}_{2}=\lambda \left(x_{2}-x_{1}^{2}\right).$$

For λ<μ<0, the system exhibits a slow attracting manifold given by $x_{2}=x_{1}^{2}$. It is possible to augment the state *x* with the nonlinear measurement $g=x_{1}^{2}$, to define a three-dimensional Koopman invariant subspace. In these coordinates, the dynamics become linear:

(A2) $$\frac{d}{dt}\left[\begin{array}{c} y_{1}\\ y_{2}\\ y_{3} \end{array}\right]=\left[\begin{array}{ccc} \mu & 0 & 0\\ 0 & \lambda & -\lambda \\ 0 & 0 & 2\mu \end{array}\right]\left[\begin{array}{c} y_{1}\\ y_{2}\\ y_{3} \end{array}\right]\;\mathrm{for}\;\left[\begin{array}{c} y_{1}\\ y_{2}\\ y_{3} \end{array}\right]=\left[\begin{array}{c} x_{1}\\ x_{2}\\ x_{1}^{2} \end{array}\right].$$

For this nonlinear system, our theory hub in Table 1 is still relevant, using

$$A=\left[\begin{array}{ccc} \mu & 0 & 0\\ 0 & \lambda & -\lambda \\ 0 & 0 & 2\mu \end{array}\right],x=\left[\begin{array}{c} x_{1}\\ x_{2}\\ x_{1}^{2} \end{array}\right].$$

Thus, it is possible that the methods we have determined a transformer uses to model the simple harmonic oscillator extend to other, more complex systems.

## Appendix C. Undamped Harmonic Oscillator Appendices

**Data generation for the undamped harmonic oscillator.** We generated 5000 sequences of 65 timesteps for various values of $\omega_{0},\Delta t,x_{0},$ and $v_{0}$. We ranged $\omega_{0}=U\left[\frac{\pi }{4},\frac{5\pi }{4}\right]$, $\Delta t=U[0,\frac{2\pi }{\omega_{0}}]$, $x_{0},v_{0}=U\left[-1,1\right]$. The undamped harmonic oscillator is periodic, so using a larger Δt was not useful. We also generated an out-of-distribution test set with $\omega_{0}=U\left[0,\frac{\pi }{4}\right]+U\left[\frac{5\pi }{4},\frac{3\pi }{2}\right]$ with the same size as the training set.

**Additional results for undamped harmonic oscillator.** In Figure A4, we see that the models were able to learn the undamped harmonic oscillator in-context, even for values of $\omega_{0}$ out of the distribution that these models were trained on. We also plotted the evolution of the encodings for our various methods on the best-performing undamped model in Figure A5. We found that our choice of *j* for the Taylor expansion method was mostly irrelevant for j≤5 in Figure A6. We also generated synthetic hidden states from the matrix exponential intermediate, and we found that the values for criterion 1,3 for the other two methods were potentially byproducts of the matrix exponential in Figure A8, giving additional correlational evidence that the matrix exponential was the dominant method of the transformer.

## Appendix D. Investigating the Damped Harmonic Oscillator (*γ* > 0)

### Appendix D.1. Mathematical Setup

The damped harmonic oscillator has three well-studied modes: underdamped, overdamped, and critically damped cases. The underdamped case occurs when $\gamma <\omega_{0}$, and it represents a spring oscillating before coming to rest. The overdamped case occurs when $\gamma >\omega_{0}$, and it represents a spring immediately returning to equilibrium without oscillating. The analytical equations for both cases are

$$\begin{array}{cc} \mathrm{Underdamped}\;(\gamma <\omega_{0}) & \mathrm{Overdamped}\;(\gamma >\omega_{0})\\ \begin{array}{cc} x_{k} & =e^{-k\gamma \Delta t}(x_{0}\cos\left(k\omega \Delta t\right)+\\ \; & \;\frac{v_{0}+\gamma x_{0}}{\omega }\sin\left(k\omega \Delta t\right))\\ v_{k} & =e^{-k\gamma \Delta t}(v_{0}\cos\left(k\omega \Delta t\right)-\\ \; & \;\left(\frac{v_{0}+\gamma x_{0}}{\omega }\gamma +\omega x_{0}\right)\sin\left(k\omega \Delta t\right)) \end{array} & \begin{array}{cc} x_{k} & =\frac{e^{-k\gamma \Delta t}}{2}(\left(x_{0}+\frac{v_{0}+\gamma x_{0}}{\omega }\right)e^{k\omega \Delta t}+\\ \; & \;\left(x_{0}-\frac{v_{0}+\gamma x_{0}}{\omega }\right)e^{-k\omega \Delta t})\\ v_{k} & =\frac{e^{-k\gamma \Delta t}}{2}(\left(\omega -\gamma \right)\left(x_{0}+\frac{v_{0}+\gamma x_{0}}{\omega }\right)e^{k\omega \Delta t}-\\ \; & \;\left(\omega +\gamma \right)\left(x_{0}-\frac{v_{0}+\gamma x_{0}}{\omega }\right)e^{-k\omega \Delta t}) \end{array} \end{array}$$

where $\omega =\sqrt{|\gamma^{2}-\omega_{0}^{2}|}$. Note that the critically damped case ($\gamma =\omega_{0}$) is equivalent to $\lim_{\gamma \to \omega_{0}^{-}}$ of the underdamped case and $\lim_{\gamma \to \omega_{0}^{+}}$ of the overdamped case. Thus, we focused our study on the underdamped and overdamped cases, and we visualize sample trajectories of both in Appendix Figure A9.

### Appendix D.2. Computational Setup for the Damped Harmonic Oscillator

We used an analogous training setup for the undamped harmonic oscillator. We generated 5000 sequences of 32 timesteps for various values of $\omega_{0},\gamma ,\Delta t,x_{0},$ and $v_{0}$ for both the underdamped and overdamped cases. For the underdamped and overdamped cases, we ranged $\omega_{0}=U\left[0.25\pi ,1.25\pi \right]$ and $\Delta t=U[0,\frac{2\pi }{13\omega_{0}}]$. We used this sequence length and bound on Δt to account for the periodic nature of the damped harmonic oscillator and also to ensure that the system did not decay to 0 too fast. For the underdamped case, we took $\gamma =U[0,\omega_{0}]$, and for the overdamped case, $\gamma =U[\omega_{0},1.5\pi ]$. We also generated an out-of-distribution test set following a similar process but using $\omega_{0}=U\left[0,0.25\pi \right]+U\left[1.25\pi ,1.5\pi \right]$.

In Figure A9, we find that a transformer trained on underdamped data was able to generalize to overdamped data with only in-context examples. This is a surprising discovery, since a human physicist who is only exposed to underdamped data would model it with the analytical function in Appendix D.1. But this method would not generalize: the underdamped case uses exponential and trigonometric functions of γΔt and ωΔt, respectively, while the overdamped case consists solely of exponential functions. We propose that our “AI Physicist” is able to generalize between underdamped and overdamped cases because it is using numerical methods that model the underlying dynamics shared by both scenarios.

### Appendix D.3. Criteria Are Less Aligned for the Underdamped Harmonic Oscillator

We evaluated all the methods for the underdamped harmonic oscillator, based on our criteria, we summarized the evaluations in Table 2, and we show the relevant figures for criteria 1, 2, and 3 in Figure A10, Figure A11 and Figure A12, respectively. While we see moderate correlational and some causal evidence for our proposed methods, we note that there was a steep drop-off across criteria between the undamped and underdamped cases. We have identified a few possible explanations for this discrepancy:

**The transformer was using a method outside of the hypothesis space.** Because the intermediates explained so little of the hidden states even when combined (Figure A12), we hypothesize that the transformer discovered a novel numerical method or was using another known method outside of our proposed hypothesis space. This was more likely for the damped case, because we decreased the range on Δt to avoid decay, which made approximate numerical solutions more accurate. But why, we asked, would it be doing this for the damped case and not for the undamped case? For our damped experiments, we decreased the range on Δt so that the trajectory did not decay to 0 too quickly, but this also allowed for approximate numerical methods to be more accurate, as demonstrated by the competitive performance of the linear multistep method with the matrix exponential method in Table 2. So, it is possible that our transformer was relying on another numerical method outside of our hypothesis space.

**Natural decay required less “understanding” by the transformer.** As the context length increased, damping forced the system to naturally decay to 0, so the transformer could use less precise methods to predict the next timestep. In Appendix Figure A13, we see that the intermediates’ encodings accordingly decayed with context length, which possibly explains the underdamped case’s diminished metrics.

**More data for the transformer to encode.** With a non-zero damping factor γ, the intermediates we investigated in Table 1 had more non-constant values in their 2×2 matrices in the damped/undamped case: the linear multistep method had 3/2 values, the Taylor expansion method had 4/2 values, and the matrix exponential method had 4/3 unique values. The increased number of non-constant values could potentially make it more difficult to properly encode intermediates.

We leave the problem of understanding the damped harmonic oscillator to future work with intermediates.
